# The Bed Nucleus of the Stria Terminalis, Homeostatic Satiety, and Compulsions: What Can We Learn From Polydipsia?

**DOI:** 10.3389/fnbeh.2019.00170

**Published:** 2019-08-01

**Authors:** Tomek J. Banasikowski, Emily R. Hawken

**Affiliations:** ^1^Department of Psychiatry, Queen’s University, Kingston, ON, Canada; ^2^Providence Care Hospital, Kingston, ON, Canada

**Keywords:** obsessive compulsive disorder, schizophrenia, dopamine, BNST, orbitofrontal cortex

## Abstract

A compulsive phenotype characterizes several neuropsychiatric illnesses – including but not limited to – schizophrenia and obsessive compulsive disorder. Because of its perceived etiological heterogeneity, it is challenging to disentangle the specific neurophysiology that precipitates compulsive behaving. Using polydipsia (or non-regulatory water drinking), we describe candidate neural substrates of compulsivity. We further postulate that aberrant neuroplasticity within cortically projecting structures [i.e., the bed nucleus of the stria terminalis (BNST)] and circuits that encode homeostatic emotions (thirst, hunger, satiety, etc.) underlie compulsive drinking. By transducing an inaccurate signal that fails to represent true homeostatic state, cortical structures cannot select appropriate and adaptive actions. Additionally, augmented dopamine (DA) reactivity in striatal projections to and from the frontal cortex contribute to aberrant homeostatic signal propagation that ultimately biases cortex-dependent behavioral selection. Responding becomes rigid and corresponds with both erroneous, inflexible encoding in both bottom-up structures and in top-down pathways. How aberrant neuroplasticity in circuits that encode homeostatic emotion result in the genesis and maintenance of compulsive behaviors needs further investigation.

## Introduction

Compulsivity can be a dominant and debilitating clinical feature of several psychiatric conditions including obsessive-compulsive disorder (OCD), schizophrenia, substance abuse, and other obsessive-compulsive spectrum disorders (OCSD) (Hariprasad et al., 1980; Berman et al., 1995; Everitt and Robbins, 2005). Compulsions are repetitive, apparent, and purposeful behaviors that are performed according to certain rules or in a stereotyped fashion. When expressed, compulsions are time-consuming, cause significant distress, and interfere in both function and quality of daily life (American Psychiatric Association, 2013). Despite an emergent body of research, identifying the neurophysiology of compulsivity is challenging and the underlying neural mechanisms of compulsivity remain speculative.

Behavioral heterogeneity and symptom dimensionality observed across compulsive spectrum disorders complicates the search for a neurological trace of compulsivity. For instance, the “classical” compulsive behaviors seen in OCD are characterized by excessive repetition of intentional “normal” behaviors and/or mental acts in an attempt to soothe discomfort brought on by obsessions, the accompanying diagnostic feature of OCD. However, not all observed compulsions captured by this definition are associated with an OCD diagnosis. Primary polydipsia, or excessive, non-regulatory water drinking, is just one example of the clinical heterogeneity found in OCSD, a behavior that shares some of its core features with other psychiatric diagnoses including schizophrenia. By isolating and identifying common neurological substrates across diagnoses marked by compulsive behaving (including primary polydipsia associated with schizophrenia and OCD) we can highlight unique neurological features specific to compulsivity in psychiatric illness.

**FIGURE 1:**
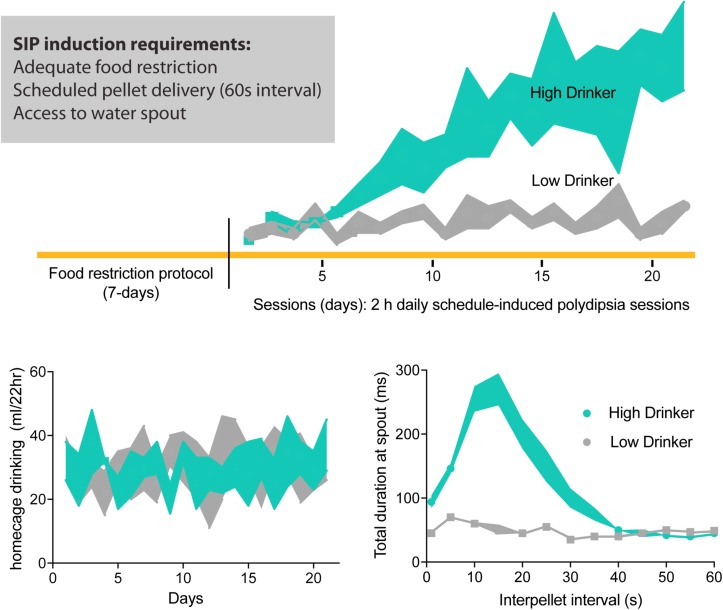
Schedule-induced polydipsia (SIP) protocol. Food restricted rodents are placed into an operant chamber with intermittent pellet delivery (recommended, 1 min interval) and access to water drinking spout. Over 2 h daily SIP sessions, some animals will develop SIP expressed as increased sessional drinking across days; low-drinking animals maintain low levels of water consumption. Daily 22 h homecage drinking during SIP protocol does not differ between high- and low-drinkers. Furthermore, high-drinkers show an adjunctive pattern of drinking during the interpellet interval 60 s, where more time is spent at the drinking spout post-prandial or adjacent to pellet consumption. Adapted with permission from Hawken et al. (2013b).

To further characterize the neuropathophysiology of psychiatric disease states, we need appropriate animal models. Some preclinical models of compulsivity have been enormously useful in distilling discrete mechanisms and neural representations of pathological behavior (Everitt and Robbins, 2005; Szechtman et al., 2017). Among the currently available animal models, schedule-induced polydipsia (SIP) is recognized as the most robust and replicable preclinical model of compulsivity (Woods et al., 1993; Moreno and Flores, 2012; Gardner Gregory, 2018). Excessive water drinking (polydipsia) occurs experimentally when hungry animals are exposed to intermittent/scheduled access to food and unlimited access to water (see Figure 1). In this protocol, some animals will drink themselves into a water intoxicated state mimicking primary polydipsia. SIP is an ideal animal model to study compulsivity specifically because the behavior is both ethological and ecological and can be induced across species, including humans (Schuster and Woods, 1966; Falk, 1969; Kachanoff et al., 1973; Dale, 1979).

Decades of research using the SIP protocol enables a full exploration of the hypothesis presented here, that disordered neuroplasticity underlying essential homeostatic emotions within limbic and cortical structures contributes to the compulsive phenotype observed in polydipsia. Because compulsive drinking (expressed both as SIP in an OCD-like animal model and also as primary polydipsia associated with schizophrenia) can be induced in humans as well as rodents, we further speculate that identified neural substrates [including the bed nucleus of the stria terminalis (BNST)] can apply to psychiatric populations, i.e., those with OCSD. Thus, herein, we synthesize the current understanding of the neurophysiology of compulsivity through the lens of polydipsia.

## Homeostatic Emotions Drive Appropriate Action Selection for Adaptive Behaving

Observationally, polydipsia is simply the overconsumption of fluid, and most often, water. Polydipsia can occur for several physiological reasons, for instance, in response to exaggerated thirst that results from disease states like diabetes insipidus. However, primary polydipsia or “psychogenic” polydipsia is non-physiologic and non-regulatory in nature, i.e., no underlying physiological condition can explain the individual’s need to overdrink. Polydipsia is maladaptive as over-drinking individuals seek and consume water despite the risk of severe life-threatening consequences (Hawken et al., 2009). Additionally, psychogenic polydipsia most commonly co-occurs with disordered thinking associated with a psychotic state. While some patients report drinking in response to excessive thirst, more report drinking excessively to feel better, to cleanse or purify themselves, or to appease/soothe other obsessions and/or delusions (Illowsky and Kirch, 1988; Millson et al., 1992). Observationally, in overdrinking patients normal thirst and satiety signals are no longer tied to homeostatic fluid regulation.

Maintaining adequate hydration is essential to an animal’s survival. Although most of our everyday drinking behavior is social, prandial, or habitual in nature and not in response to thirst, multiple fail-safe systems are in place to ensure that fluids lost are quickly replenished (McKinley et al., 2019). One such system is our brain’s ability to accurately recognize the feeling or emotion of “thirst” and behave appropriately (drink) to satiation. Most studies regulating thirst indicate that normal drinking behavior is generally preparatory, and to a lesser extent consummatory, in nature (Konorski, 1967; Egan et al., 2003; Saker et al., 2016). In other words, the brain quickly adjusts behavior in anticipation of falling dangerously far from homeostasis by initiating actions to maintain or restore fluid balance. Within the preparatory/consummatory framework, normal ingestive drinking behavior is therefore tightly regulated by a combination of incentive and homeostatic cues making the drive to drink both introceptively driven yet also perpetuated by anxiogenic situations that are extroceptively mediated. Drinking when thirsty is both pleasurable and rewarding, and extinction of thirst activates dopamine (DA) pathways in the brain (Ettenberg and Camp, 1986). Thus, many of the same brain regions that are activated in decision making, reward, stress, fear, and anxiety also mediate water seeking and intake (Egan et al., 2003; Shin and Liberzon, 2010). Such a coordinated response by multiple interconnected circuits across the brain ensures specific goal-directed behaviors are engaged to maintain fluid homeostasis to promote survival (Allen et al., 2019).

Being entirely subjective, thirst can be categorized along with the undeniable physiological need to eat, breath, and sleep as an *essential* homeostatic “emotion.” This primal and essential emotion arises from interoceptive cues compiled by sensory circumventricular organs that sense dramatic shifts in water and energy homeostasis (McKinley et al., 2019; Zimmerman et al., 2019). From these interfaces, interoceptive/homeostatic information is projected onto the cortex though polysynaptic relays including structures in the limbic system like the BNST (McKinley et al., 2019). Cortical structures decode the ascending homeostatic information to motivate appropriate behavior (to drink) and restore physiological thresholds, or quench one’s thirst (Craig, 2003). Interestingly, satiety or satiation of thirst is likely more than a lack of thirst or removal of a thirsty state, as distinct cortical regions are activated in each condition (McKinley et al., 2019). Allen et al. (2019) recently reported thirsty and sated states produce separate but proximal patterns of neuronal activation across several structures in the rodent brain. Such a brain-wide and independent representation of drinking behavior likely reflects the fact that thirst-satiety signals are evolutionarily necessary to prevent the threat of excessive hydration. Indeed, the satiety signal is so important that swallowing water is perceived first before any systemic absorption of water (McKinley, 2009). Thus satiety, like thirst, is itself an essential homeostatic emotion, with both playing pivotal roles in preventing the dangerous physiological consequences of unregulated non-homeostatic drinking.

While thirst and satiety are likely represented in various iterations across the brain, imaging studies in humans infused intravenously with hypertonic saline to induce thirst identify key structures involved in the conscious detection or “feeling” of thirst. Both positron emission tomography (PET) and functional magnetic resonance imaging (fMRI) show that the anterior cingulate (ACC) and insular cortex are activated in subjects made thirsty, and that satiation of thirst by drinking quickly extinguished that activity (Denton et al., 1999a,b; de Araujo et al., 2003; Egan et al., 2003). Upon wetting the mouth, activation of orbitofrontal cortex (OFC) occurs, a feature also extinguished with satiety. If subjects are asked to overdrink, a reactivation of insular cortex is seen, along with activation of the amygdala and periaqueductal gray regions – regions implicated in the “valence” networks and associated emotions including stress, fear, and anxiety (Saker et al., 2016). Despite widespread activity in several cortical and other brain regions correlating with thirst scores and subsequent satiety, conscious feelings of “thirst” and “satiety” and other homeostatic emotions arise from a yet to be identified aggregate or pattern of circuit/network activity (Allen et al., 2019).

Nevertheless, cortical regions including the ACC, insular, and OFC cortices have been implicated in many homeostatic emotions beyond thirst, including hunger and deep pain, reflecting an adverse condition within the body that requires immediate behavioral response (Craig, 2003). Generally, the ACC is activated by occurrences of adverse conditions that require decision making as to appropriate strategic responding (Gehring and Taylor, 2004). Accordingly, lesions of the ACC in humans, primates, and rats results in discernable apathy, or indifference and a lack of motivation to rectify an adverse condition (Eslinger and Damasio, 1985; Johansen et al., 2001). Conversely, a role for the insular cortex may be to identify the specific homeostatic perturbation and tag it with the appropriate emotional label – for instance, the insular cortex detects dehydration and translates it as thirst. Lastly the OFC, a cortical structure interconnected with ACC and insula, provides the hedonic component to the behavioral response of drinking to signal that water in the mouth is pleasant if thirsty but less so when not thirsty. Thus, sensory signals conveying the homeostatic state of the individual will reach the OFC, from the bottom-up, and be interpreted according to their biological significance to dictate appropriate action selection. In this proposed schema (McKinley, 2009), the cortical generation of the emotion of thirst would involve activations of neurons within the ACC for motivational intensity (urgency), the insula for homeostatic specificity (e.g., thirst, hunger, and satiety), and the orbito-frontal region for decision to initiate or maintain fluid seeking and consummatory behavior. However, as a clear neural “seat” of homeostatic emotions remains unknown, we can only speculate how homeostatic signals stop eliciting appropriate and adaptive behavioral repertoires in polydipsia.

## Psychogenic Polydipsia in Schizophrenia

In primary or “psychogenic” polydipsia, homeostatic emotions somehow become disassociated from appropriate behavioral selection. A person with polydipsia may either feel thirsty but is unable to encode satiety to stop drinking behavior or is not drinking in response to thirst in the first place. Whether compulsive over-drinking results from an altered perception of thirst and/or satiety is unconfirmed (Goldman et al., 1996). However, in response to infusion of hypertonic saline, thirst ratings in compulsive water drinkers are higher than in normal drinkers, and remain elevated following drinking episodes. Conversely, drinking rapidly abolishes thirst emotion in non-compulsive drinkers (Thompson et al., 1991). To understand how or when a disconnect between homeostatic emotional drive-states and goal-directed behaviors occur, we must examine the context in which polydipsic behavior manifests.

The significant incidence of psychogenic polydipsia in patients with severe chronic psychiatric illness (de Leon et al., 1994) highlights central traits key to resolving the neurophysiology of compulsive overdrinking. While not exclusive to mental illness (Stuart et al., 1980), polydipsia is detected in over 20% of long-term institutionalized patients with a diagnosis of schizophrenia (Blum et al., 1983; Kirch et al., 1985; de Leon et al., 1996; Mercier-Guidez and Loas, 2000). Additionally, patients with polydipsia associated with schizophrenia are also more likely to be male and to compulsively smoke (de Leon et al., 1994; Mercier-Guidez and Loas, 2000). Furthermore, episodes of polydipsia-induced water intoxication in schizophrenia are correlated with a greater severity of psychotic illness, characterized by earlier onset of psychosis, severe symptom breakthrough in periods of illness stability, higher rates of alcohol abuse, lower levels of global functioning, and longer institutionalized living (Poirier et al., 2010). From a mechanistic perspective, severe polydipsia may represent an identifiable subgroup that exhibits more neurological impairments than those without compulsive drinking tendencies (Illowsky and Kirch, 1988).

Neurodegenerative processes may underlie some of the illness progression observed in schizophrenia (Lieberman, 1999) and may contribute to the incidence of polydipsia in this population. In first episode schizophrenia, structural alterations are present indicating whole brain and hippocampal volume reductions (Steen et al., 2006; Vita et al., 2006). Importantly, some structural changes progress in a subgroup of patients during the course of illness suggesting that polydipsia could be a behavioral expression of neurodegeneration (DeLisi et al., 2004). In support of this hypothesis, primary polydipsia is significantly associated with a chronic course of psychotic illness (de Leon et al., 1996). Accordingly, when polydipsia develops it typically onsets 5–15 years following the original psychiatric diagnosis (Vieweg V. et al., 1984; Vieweg W.V. et al., 1984). With disease progression in schizophrenia, stereotypical behaviors also become increasing common and can include compulsive smoking, odd grooming patterns, pacing, and other repetitive motor actions (Arieti, 1974; Luchins et al., 1992; Alexander et al., 1993; Tracy et al., 1996; Morrens et al., 2006). Polydipsia is typically clustered into this stereotypical or “bizarre behaviors” category. Thus, some posit that stereotypies and other ritualistic mannerisms (including polydipsia) co-occurring in schizophrenia constitute a coherent group of symptoms that are mediated by common (across diagnoses) neurological abnormalities (Luchins, 1990).

## Polydipsia, Schizophrenia, and OCD: Is There Neurobiological Overlap?

In part due to polydipsia’s temporal association with other repetitive behaviors (grooming, pacing, and ruminating), some suggest that polydipsia is related to the specific stereotypies and compulsions observed in OCSD or OCD itself (Deas-Nesmith and Brewerton, 1992; Shutty et al., 1995; Subramanian et al., 2017). However, the incidence of co-occurring polydipsia in OCD is far less evident than the association of polydipsia with schizophrenia. Nevertheless, rates of obsessive-compulsive symptoms in psychotic illness vary greatly and are estimated to range between 2.5 and 64% (de Haan et al., 2013; Grover et al., 2017). Differential diagnoses pose challenges for both clinicians and investigators to distinguish between compulsions and stereotypic behavior (Berrios, 1989). Incidentally, a substantial proportion (up to 37.5%) of those with schizophrenia also have a diagnosis of OCD (Lysaker et al., 2000; Nechmad et al., 2003; de Haan et al., 2013; Grover et al., 2017). The high incidence rate of schizophrenia with concurrent OCD symptomology resulted in the formulation of a new clinical entity for the dual diagnosis termed “schizo-obsessive disorder” (Hwang and Opler, 1994; Scotti-Muzzi and Saide, 2017). Therefore, comparing the neurophysiology of compulsions associated with schizophrenia with OCD could identify common disease-specific mechanisms of compulsive behavior.

Indirect lines of evidence from imaging studies support a loss of brain matter across brain structures common to polydipsia, schizophrenia, and OCD (Table 1). While limited, imaging studies of patients with polydipsia associated with schizophrenia show a global loss of brain matter suggested by the presence of significantly enlarged ventricles as compared to those without polydipsia (Emsley et al., 1995; Leadbetter et al., 1999). While schizophrenia itself is not associated with ventricular enlargement (Ebdrup et al., 2010), individuals with OCD and poor insight also exhibit bilateral ventricular enlargement (Luxenberg et al., 1988; Okasha et al., 2000). Gray and white matter loss throughout fronto-cortico-striatal and limbic regions known to be dysfunctional in both schizophrenia and OCD could account for the observed ventricular enlargement (Harrison et al., 2009; Fornito et al., 2013; Jung et al., 2013; Huang et al., 2018).

**TABLE 1:** Gray and white matter volume changes reported across the brain in polydipsia associated with schizophrenia, schizophrenia, and obsessive compulsive disorder (OCD).

Brain region	Polydipsia in schizophrenia	**Schizophrenia**	**OCD**	**References**
Enlarged ventricles	Yes	NC	Yes^*^	Luxenberg et al., 1988; Emsley et al., 1995; Leadbetter et al., 1999; Okasha et al., 2000; Ebdrup et al., 2010
Insula cortex (Left)	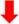	NC	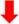	Pujol et al., 2004; van den Heuvel et al., 2009; Alvarenga et al., 2012; Nagashima et al., 2012
Orbitofrontal cortex		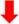 ^*^	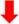 ^*^	Luxenberg et al., 1988; Goldstein et al., 1999; Gur et al., 2000; Okasha et al., 2000; Wilke et al., 2001; Shapleske et al., 2002; Kawasaki et al., 2004; Riffkin et al., 2005; Valente et al., 2005; Sapara et al., 2007; Tregellas et al., 2007; Nakamura et al., 2008; Schobel et al., 2009a; van den Heuvel et al., 2009; Alvarenga et al., 2012
Anterior cingulate cortex		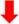		Goldstein et al., 1999; Gur et al., 2000; Wilke et al., 2001; Shapleske et al., 2002; Kawasaki et al., 2004; Riffkin et al., 2005; Tregellas et al., 2007; Schobel et al., 2009a
Hippocampus	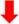 (Anterior)	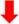 ^*^	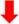 ^*^	Luchins et al., 1997; Goldman et al., 2007, 2011; Atmaca et al., 2008; Schobel et al., 2009a,b; Ebdrup et al., 2010; Wood et al., 2010; Boedhoe et al., 2017; Fouche et al., 2017; Reess et al., 2017
Parahippocampal gyrus	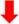	NC	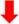 ^*^	Pujol et al., 2004; Sim et al., 2006; van den Heuvel et al., 2009; Alvarenga et al., 2012; Nagashima et al., 2012
Amygdala	NC	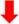	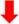 ^*^	Pantelis et al., 2003; Pujol et al., 2004; Atmaca et al., 2008;
Nucleus Accumbens		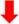		Ebdrup et al., 2010
Caudate		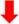	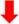	Crespo-Facorro et al., 2007; van den Heuvel et al., 2009; Ebdrup et al., 2010

NC, no change. ^*^Negative correlation with illness symptoms.

In line with a view that reduced functional neuroplasticity in key networks contributes to OCSD, structural volume loss across fronto-cortico-striatal loops is consistently reported in both schizophrenia and OCD. In schizophrenia, reduced OFC and ACC volumes are linked with greater severity of formal thought disorder, low levels of insight, and a longer duration of the illness (Goldstein et al., 1999; Gur et al., 2000; Wilke et al., 2001; Shapleske et al., 2002; Kawasaki et al., 2004; Riffkin et al., 2005; Shad et al., 2006, Shad et al., 2007; Tregellas et al., 2007; Bellani and Brambilla, 2008; Nakamura et al., 2008; Schobel et al., 2009a). Resting state connectivity studies also correlate checking behavior in OCD with alterations in frontal regions, specifically the OFC and ACC (Harrison et al., 2013). Patients with polydipsia associated with schizophrenia additionally exhibit volume reductions in the left insular cortex (Nagashima et al., 2012) as do individuals with OCD and exaggerated checking compulsions (Pujol et al., 2004; van den Heuvel et al., 2009; Alvarenga et al., 2012). In striatal structures, reduced volumes are reported in the caudate nucleus in both treatment naïve, first-episode patients with schizophrenia and in OCD (Pujol et al., 2004; Crespo-Facorro et al., 2007; Atmaca et al., 2008; Rotge et al., 2009; van den Heuvel et al., 2009; Ebdrup et al., 2010). Additionally, in treatment refractory OCD, amygdalar volumes are negatively correlated with symptom “harm/checking” scores, illness duration, and symptom-severity (Pujol et al., 2004; Atmaca et al., 2008; Boedhoe et al., 2017; Fouche et al., 2017; Reess et al., 2018). Thus, reduced functional capacity of fronto-cortico-striatal and limbic structures likely contribute to the development of specific maladaptive behaviors associated with each disease state. However, how compulsive drinking (polydipsia) reflects deficits in neuroplasticity within pathologically common structures cannot be determined from available human imaging studies. Further insight into the neural plastic mechanisms regulating polydipsia can be gained from pre-clinical animal models of compulsive drinking.

## Dopamine Drives Polydipsia in Humans and Rodents

Cumulative evidence suggests that schedule-induced polydipsia (SIP) is a valid animal model of compulsive water drinking (Woods et al., 1993; Moreno and Flores, 2012; Gardner Gregory, 2018). Good disease models show a similar pattern of symptoms to the disease being modeled, have measures that can be objectively quantified, and are both reproducible and robust (Geyer and Markou, 1994). In rodents, excessive drinking in the SIP protocol was first observed (Falk, 1961) when a hungry animal consumed excessive amounts of water during predictable but intermittent food access (i.e., scheduled; Figure 1). Like primary polydipsia, compulsive drinking induced by the SIP protocol is non-homeostatic in that animals engage in drinking behavior in the face of excessive overhydration (Falk, 1971). Like compulsions, SIP behavior is excessive, ritualistic, and maladaptive as animals can drink themselves into a dilutional water-toxic state that results in death (Hawken et al., 2013b). Furthermore, not all psychiatric patients nor all animals exposed to the SIP paradigm develop compulsive drinking. Such face validity suggests SIP is a powerful pre-clinical tool to help us understand how neurological, environmental, and genetic factors trigger or contribute to this compulsive phenotype (Rosenzweig-Lipson et al., 2007; Toscano et al., 2008; Moreno and Flores, 2012).

As the compulsion to overdrink in humans is postulated to be a behavioral manifestation of schizophrenia, some of the discrete neurobiology underlying the expression of polydipsia can be inferred from the neuropathology of schizophrenia itself. Currently, the etiology of schizophrenia continues to be debated, although it is generally conceptualized as a complex neurodevelopmental disorder with features of neurodegeneration and risk factors (both genetic and environmental) predicting its onset (Lieberman, 1999; Lewis and Lieberman, 2000; Tandon et al., 2008a,b). Historically, increased striatal DA function and impaired prefrontal cortical activity are the two most robust neuropathological features associated with schizophrenia’s core positive, negative, and cognitive symptoms (Lewis and Anderson, 1995; Laruelle and Abi-Dargham, 1999). Accordingly, DA is identified as one of the key neuromodulators dysregulated in schizophrenia.

In its original inception, the DA hypothesis proposed that schizophrenia results from DA overactivity within subcortical striatal structures (van Rossum, 1966). Evidence for hyperdopaminergia in schizophrenia was initially observed in chronic users of psychostimulants, where excessive use of DA-augmenting drugs induced psychotic states (Bell, 1965; Angrist and Gershon, 1970). Concurrently, antipsychotic drugs developed to alleviate psychosis did so through selectively blocking the DA D2 receptors (Seeman et al., 1975). Much later, increased dopaminergic activity in schizophrenia was confirmed by imaging studies. In patients with schizophrenia, displacement of DA at D2 receptors by amphetamine (AMPH)-induced DA release is increased in the striatum (Laruelle et al., 1996; Abi-Dargham et al., 1998; Laruelle and Abi-Dargham, 1999). Additionally, estimated striatal DA release following AMPH correlates with the severity of AMPH-exacerbated psychotic symptoms (Laruelle et al., 1999). However, striatal hyperdopaminergia fails to fully explain the negative and cognitive symptoms that also characterize schizophrenia. Thus, in a revised DA hypothesis, hyperdopaminergia, or increased reactivity of the mesolimbic DA system, is now primarily linked to the positive, psychotic features of schizophrenia, i.e., hallucinations and delusions (Carlsson, 1974) while a hypodopaminergia of the PFC may underlie observed cognitive dysfunction (Slifstein et al., 2015).

Psychogenic polydipsia in schizophrenia is temporally tied to psychosis, arguably a state of striatal hyperdopaminergia, where patients’ increased drinking behavior parallels psychotic exacerbation and is normalized when psychosis remits (Raskind et al., 1975; Zubenko et al., 1984). This suggests a role for elevated striatal DA function in primary polydipsia associated with schizophrenia. In animals, an ideally fluctuating amount of striatal DA is necessary for compulsive drinking in SIP to develop. Fixed, short intervals of food presentation to a food-restricted animal stimulates necessary DA activity (Cousins et al., 1999) with more DA released in the nucleus accumbens (NAc) following food consumption when animals are hungry versus when they are sated (Ahn and Phillips, 1999). In a hungry rat, DA levels increase in the NAc along-side elevated drinking over the course of several SIP trials (Hooks et al., 1994; Weissenborn et al., 1996; Moreno and Flores, 2012). This implies that an appropriately reactive DA system is necessary for SIP. Disrupting the integrity of the short-latency DA signal with acutely administered DA agonists and antagonists prior to each SIP session consistently prevents SIP development (see Table 2) (Mittleman et al., 1994; Flores and Pellón, 1995; Escher et al., 2006; López-Grancha et al., 2008). Furthermore, increased D2-like and decreased D1-like receptor binding throughout the NAc, medial prefrontal cortex, amygdala, and the ventral tagmental area (VTA) are associated with SIP expression (Pellón et al., 2011). Therefore, increased dopaminergic tone and/or reactivity throughout the mesolimbic and mesocortical networks may lead to aberrant reward-related learning (Banasikowski et al., 2012) that facilitates the compulsive water consumption as seen in primary polydipsia.

**TABLE 2:** Interventions and effects on compulsive behaving induced by the schedule-induced polydipsia (SIP) protocol.

Intervention	**Route – Region**	Drug-Dose mg/kg	Time pre-SIP/ acquisition/ expression	SIP effect (↑↓)	**References**
DA agonist	Systemic	Amphetamine	Acquisition		
		0.25		↓	Sanger, 1977
		0.5		↓	Sanger, 1977; Didriksen and Christensen, 1994
		1.0		↓	Yoburn and Glusman, 1982
		2.0		↓	Sanger, 1977
			Expression		
		0.5		↓	Kuribara and Tadokoro, 1980; López-Grancha et al., 2008
		1.0		↓ no effect	McMillan, 1979; Sanger and Corfield-Sumner, 1979; Kuribara and Tadokoro, 1980; Robbins et al., 1983; Williams and White, 1984; Flores and Pellón, 1997; Shen et al., 2001
		1.5		↓	Wayner et al., 1973
		2.0		↓	Wayner et al., 1973; Sanger and Corfield-Sumner, 1979; Kuribara and Tadokoro, 1980; Robbins et al., 1983; Pellon and Blackman, 1992; Mittleman et al., 1994; Flores and Pellón, 1995; Shen et al., 2001; López-Grancha et al., 2008
		3.0		↓	McMillan, 1979; Yoburn and Glusman, 1982; Williams and White, 1984
		4.0		↓	Flores and Pellón, 1995
		10		↓	Williams and White, 1984
			Pre-SIP		
		1.5		↑	Hawken and Beninger, 2014
		5		↑	Mittleman and Valenstein, 1985
		apomorphine	Acquisition		
		0.05, 0.5, 1.0		↓	Snodgrass and Allen, 1988
			Expression		
		0.025, 0.1		↓	Robbins et al., 1983
		0.7, 1.3		↓	Snodgrass and Allen, 1988
		Quinpirole	Expression		
		0.025, 0.05, 0.1, 0.2		↓	Mittleman et al., 1994
		SKF 38393	Expression		
		4.0, 8.0		↓	Mittleman et al., 1994
		SKF 82958	Expression		
		0.02, 0.04, 0.08,0.16		↓	Mittleman et al., 1994
		SKF 83566	Expression		
		0.25, 0.5, 1.0		↓	Mittleman et al., 1994
		Cocaine	Expression		
		10, 20		↓	Jones et al., 1994; López-Grancha et al., 2008
	Intra NAc, PFC	12.5, 25, 50, 100 μg			Jones et al., 1994
	Systemic	Phenylethylamine	Expression		
		10, 20		↓	Mittleman et al., 1993
		RO5263397 TAAR-1	Expression		
		3.0, 6.0, 10.0		↓	Sukhanov et al., 2019
DA antagonist	Systemic	Raclopride	Acquisition		
		0.05, 0.1		↓	Didriksen and Christensen, 1994
		Raclopride	Expression		
		0.05, 0.1		↓	Didriksen and Christensen, 1993
		0.05, 0.15, 0.5		↓	Ryan et al., 1993
		Haloperidol	Acquisition		
		0.01		↓	Didriksen and Christensen, 1994
		Haloperidol	Expression		
		0.1, 0.2, 0.3		↓	Snodgrass and Allen, 1987
		0.25, 0.5, 0.75, 1.0		↓	Keehn et al., 1976; Keehn and Riusech, 1977
		0.2, 0.8		↓	Todd et al., 1992
		0.32		↓	Mittleman et al., 1994
		Cis(z)-flupentixol	Acquisition		
		0.05		↓	Didriksen and Christensen, 1994
		Sch 23390	Acquisition		
		0.005, 0.01, 0.025		↓	Didriksen and Christensen, 1994
		Sch 23390	Expression		
		0.025, 0.05		↓	Didriksen et al., 1993
		0.01, 0.02		↓	Todd et al., 1992
		0.8		↓	Mittleman et al., 1994
		Clozapine	Acquisition		
		10		↓	Didriksen and Christensen, 1994
		Clozapine	Expression		
		10		↓	Didriksen et al., 1993
		Sertindole	Acquisition		
		1.25		↓	Didriksen and Christensen, 1994
		Spiperone	Acquisition		
		0.06, 0.125		↓	Porter et al., 1984
		Risperidone	Acquisition		
		0.2, 0.4		↓	Didriksen, 1995
		Pimozide	Acquisition		
		0.5, 1.0		↓	Porter et al., 1984
		1.0		↓	Snodgrass and Allen, 1989
		Chlorpromazine	Expression		
		5.0, 10.0		↓	Canon and Lippa, 1977
		0.5, 1.0, 2.0		↓	Kuribara and Tadokoro, 1980
5 HT agonist	Systemic	Fluoxetine	Expression		
		3.0		↓	Martin et al., 2002; Rodriguez et al., 2017
		5.0		↓	Woods et al., 1993
		6.5		↓	Hogg and Dalvi, 2004
		10.0		↓	Martin et al., 2002; Prus et al., 2015; Rodriguez et al., 2017
		20.0		↓	Rodriguez et al., 2017
		30.0		↓	Martin et al., 2002; Prus et al., 2015
		Clomipramine	Expression		
		5.0		↓	Woods et al., 1993
		16.0, 22.0, 30.0		↓	Rodriguez et al., 2017
		Desipramine	Expression		
		5.0		↓	Woods et al., 1993
		Citalopram	Expression		
		0.3, 1.0, 3.0		↓	Navarro et al., 2015
		8-OH-DPAT	Expression		
		0.1, 1.0		↓	Ryan et al., 1993
		10		↓	Woods-Kettelberger et al., 1996
		DOI	Expression		
		0.1, 0.3, 0.5		↓	Navarro et al., 2015
		0.1, 0.5, 1.0		↓	Lu et al., 1992
		RO 60-0175	Expression		
		0.3, 1.0, 3.0		↓	Martin et al., 2002
		1.0, 3.0, 10.0		↓	Rodriguez et al., 2017
		MCPP	Expression		
		3.0		↓	Rodriguez et al., 2017
		Buspirone	Expression		
		3.0, 10.0		↓	Ryan et al., 1993
		Ipsapirone	Expression		
		3.0, 10.0		↓	Ryan et al., 1993
5 HT antagonist	Systemic	Ritanserin	Expression		
		2.5		↑	Lu et al., 1992
		SB 242084	Expression		
		0.1,0.3,1		↑	Martin et al., 2002
		1.0, 2.0		↑	Hogg and Dalvi, 2004
		amperozide	Expression		
		1.6, 2.0, 4.0		↓	Tung et al., 1994
5 HT depletion	Diet		Expression	↑	Merchán et al., 2017
NE agonist	Systemic	Atomoxetine	Expression		
		1.0		↓	Ansquer et al., 2014
		Duloxetine	Expression		
		30.0, 100.0		↓	Prus et al., 2015
		Bespiridine	Expression		
		10.0		↓	Woods-Kettelberger et al., 1996
	Lateral hypothalamus	Norepinephrine	Acquisition	↓	Singer et al., 1975
NE depletion		DSP-4	Pre-SIP		
		50.0		↓	Lu et al., 1992
Lesion or systemic/ intracerebralventricular injection i.c.v)	Anterior insular cortex	Quinolinic acid	Acquisition Expression	↓ ↓	Belin-Rauscent et al., 2016
	Frontal cortex		Acquisition	↓	Bigler et al., 1974
	Dorsal hippocampus	Aspiration	Acquisition	↓	Mittleman et al., 1990
	hippocampus		Acquisition	↑	Devenport, 1978
	Dorsal lateral striatum	6-OHDA	Acquisition	No effect	Mittleman et al., 1990
	Lateral septum	6-OHDA	Acquisition	↑	Taghzouti et al., 1985
	septum	Radiofrequency thermal electrode	Expression	↑	Wayner and Greenberg, 1972
	NAc core	6-OHDA	Acquisition	↓	Robbins and Koob, 1980; Wallace et al., 1983; Mittleman et al., 1990
		NMDA-induced	Acquisition	↓	Weissenborn et al., 1996
		Ibotenic acid	Acquisition	↓	Annett and Robbins, 1987
		6-OHDA	Expression	↑	Robbins et al., 1983
	Locus coeruleus	Radiofrequency thermal electrode	Expression	↓	Lu et al., 1992
	VTA	Radiofrequency thermal electrode	Expression	↓	Lu et al., 1992
	Lateral hypothalmus	NMDA-induced	Acquisition	↑	Winn et al., 1992
	Zona incerta	Electric current	Acquisition	↓	Roehrs and Allen, 1980
	Adrenalectomy		Acquisition	↑ ↓	Devenport, 1978; Mittleman et al., 1992
	Adrenalectomy/ Demedullation		Expression	↓	Wright and Kelso, 1981
	Systemic	Corticosterone	Acquisition		
		200.0		↑	Cirulli et al., 1994
		Dexamethasone	Acquisition		
		0.4		↑	Levine and Levine, 1989
		FG 7142	Acquisition		
		1.0		↑	Mittleman et al., 1988a
		3.0		↓	Mittleman et al., 1988a
		FG 7142	Expression		
		3.0, 5.7, 9.0		↓	Mittleman et al., 1988a
	i.c.v.	CRF	Expression		
		0.1, 0.5 μg		↓	Cole and Koob, 1994
	Systemic	RO 15-1788	Expression		
		10.0		↓	Mittleman et al., 1988a
GABA agonist	Systemic	Diazepam	Acquisition		
		1.0		↑	Mittleman et al., 1988a
		Diazepam	Expression		
		0.25		↑	Kuribara and Tadokoro, 1980
		0.5		↑	Pellon and Blackman, 1992
		1.0		↑	López-Grancha et al., 2008
		2.0		↑	Pellon and Blackman, 1992
		3.0		↓	Mittleman et al., 1988b, 1994; López-Grancha et al., 2008
		5.0		↓	Mittleman et al., 1988b; López-Grancha et al., 2008
		Chlordiazepoxide	Expression		
		2.0		↑	Lobarinas and Falk, 1998
		10.0, 20.0		↓	Sanger and Corfield-Sumner, 1979
		Pentylenetetrazol	Expression		
NMDA antagonist	Systemic	MK-801	Pre-SIP		
		0.5		↑	Hawken et al., 2011
		Amantadine	Expression		
		40.0, 60.0		↓	Escher et al., 2006
		20.0		↓	López-Grancha et al., 2008
DBS	NAc	0.5 mA	Expression	↓	van Kuyck et al., 2008
	Mediodorsal thalamus	0.5 mA	Expression	↓	van Kuyck et al., 2008
	BNST	0.2 mA	Expression	↓	van Kuyck et al., 2008
Footshock		2.5 mA −(0.5s) × 180	Pre-SIP	↓	Brett et al., 1982
		Mild	Expression	↑	Segal and Oden, 1969
		0.1 mA	Expression	↑	Galantowicz and King, 1975
		1.0 mA	Expression	No effect	Galantowicz and King, 1975
		2.0 mA	Expression	↓	Galantowicz and King, 1975

By inducing subtle but functionally significant augmentation of the DA system, compulsive drinking can be enhanced. We assessed the ability of amphetamine sensitization (subchronic AMPH), NMDA hypofunction (subchronic MK-801), and social isolation (from weaning) models of schizophrenia-like symptoms to augment striatal DA and “mimic” a schizophrenia-like state in rodents. Evidence suggests that repeated AMPH and MK-801 permanently increases DA transmission along the rat VTA-NAc pathway (Hall et al., 1998; Jentsch et al., 1998) (for review see Svensson, 2000; Lodge and Grace, 2008, 2012; Beninger et al., 2009). Chronic exposure to NMDA induces a loss of GABAergic transmission to disinhibit DA neuron population activity of midbrain DA neurons (Floresco et al., 2003; Lodge and Grace, 2006). Cortical parvalbumin-positive GABA interneurons affected by chronic exposure to NMDA receptor antagonists are similarly affected by isolation rearing, a developmental model of schizophrenia (Jones et al., 1990; Wilkinson et al., 1994; Hall et al., 1998; Heidbreder et al., 2000; Miura et al., 2002; Fabricius et al., 2010, 2011; Han et al., 2011; Powell et al., 2012). As predicted, all models that increase striatal DA reactivity also significantly increased compulsive water drinking expressed as SIP (Hawken et al., 2011, 2013a; Hawken and Beninger, 2014).

Collectively, both animal and human data suggest that those with augmented, but intact, DA systems are prone to develop compulsive polydipsic behavior. In SIP, the temporal contiguity of the DA signal evoked by food stimuli precipitates a non-specific locomotor/approach behavior to an available drinking spout and facilitates acquisition of SIP (Jacobs and Farel, 1971; Vaccarino et al., 1989; Blackburn et al., 1992; Wise, 2004; Alcaro and Panksepp, 2011). Rats with a “compulsive phenotype” may have a more reactive DA system (e.g., a larger food-evoked DA signal and subsequent locomotor activation) and thus an increased sensitivity to the “activating” properties of food and other stimuli associated with schedules inside and outside the SIP-protocol. While overall homecage drinking may not differ between rats with SIP and those without, compulsive phenotypes may demonstrate a pattern of adjunctive drinking to any predictable (scheduled) environmental cues (Hawken et al., 2013b). In schizophrenia and particularly during psychotic episodes, individuals experience an exaggerated striatal DA response when presented with reinforcing stimuli, like meals, initiating generalized behavior and possibly drinking, if the appropriate stimuli (access to water) are available. Thus, the acutely psychotic and hospitalized individual could be prone to stereotypical/ritualistic behaviors due to augmented DA reactivity and the scheduled routines of institutionalized living. How DA specifically modulates circuits and systems central to OCSD to generate compulsive actions, however, must be studied through investigating SIP as an animal model of OCD.

## Schedule-Induced Polydipsia as a Model of Obsessive Compulsive Disorder

Schedule-induced polydipsia is recognized as a valid model of compulsive behaving (i.e., OCD) in part due to the ability of serotonin reuptake inhibitors (SRIs) to disrupt SIP development and expression (Table 2). The ameliorative effects of SRIs on OCD symptoms were originally sufficient to assume serotonin (5-HT) dysfunction to be at the neurophysiological core of OCD symptoms (DeVeaugh-Geiss et al., 1989) for review see, (Barr et al., 1992). However, SRI monotherapy often fails (McDougle et al., 1994) and a subset of patients treatment-refractory to SRIs have benefited from adjunctive therapies that include DA antagonists (e.g., antipsychotics; for review see Koo et al., 2010).

Evidence to support a role for DA in OCD and other compulsive behaviors is reported. Notably, pharmacologically increasing DA neurotransmission with DA agonists exacerbates compulsivity traits and behaviors in both animal models (Szechtman et al., 1998) and susceptible humans (Frye and Arnold, 1981; Rosse et al., 1993; Kotsopoulos and Spivak, 2001). Evidence for modified synaptic DA activity (via dopamine transporter binding) in the striatum is also found in patients with OCD (van der Wee et al., 2004; Hesse et al., 2005). In SIP-prone rats, differences in the binding affinity of DA D1-like/D2-like receptors exist in limbic and cortical circuits (amygdala, VTA, NAc, and medial prefrontal cortex [mPFC]) (Pellón et al., 2011). Furthermore, pharmacological pretreatment with either DA agonists/antagonists, serotonergic or monoamine modulators, reduce SIP behaviors (i.e., drinking and licking; see Table 2) (Moreno and Flores, 2012; Navarro et al., 2015; Rodriguez et al., 2017; Sukhanov et al., 2019). High drinking rats also show elevated serotonergic activity in the amygdala (Moreno et al., 2012) and reduced 5-HT_2A_ receptor binding in the mPFC (Mora et al., 2018) with additional evidence for changes in DA activity in the PFC, NAc, and amygdala (Moreno et al., 2012). Evidence supports a clear role for 5-HT, DA, and potentially 5-HT-DA interactions in the pathophysiology of compulsive phenotypes.

The DA hypothesis in OCSD is based on region-specific DA dysfunction within cortico-limbic-striato-thalamic-cortical (CLSTC) loops (Modell et al., 1989). For instance, enhanced or attenuated DA reactivity in some neurocircuits may change the weight of circuits to bias behavior toward habitual and compulsive responding (Nelson and Killcross, 2006; Ott and Nieder, 2019). Habits result from behavior performed frequently with an unchanging outcome and once established, are generally less flexible to future changes in predicted/expected outcomes (Balleine and Dickinson, 1998; Gillan et al., 2014). The medial parts of the striatum are necessary for goal-directed, outcome-sensitive behaviors but as responding becomes habitual, neuronal control gradually shifts to more lateral parts of the striatum critical for behavioral habits (Yin et al., 2004, 2006). In these regions, DA modulates the acquisition of stimulus-outcome, action-outcome and stimulus-response associations (Yin et al., 2004, 2006; Clarke et al., 2008; Xue et al., 2008; Belin et al., 2009). Flexible and appropriate behavioral responses or “cognitive control” via the frontal cortex is also heavily influenced by dopaminergic neuromodulation (Ott and Nieder, 2019). Thus, aberrant DA functioning in striatal and cortico-striatal loops that include the OFC and mPFC are believed to promote maladaptive or excessive habit formation like that observed in psychiatric disorders (Robbins et al., 2012; Gillan et al., 2016).

Accordingly, a dysfunctional balance between goal-directed and habit behavior with an over reliance on habitual circuitry may precipitate the compulsive phenotype (Gillan et al., 2011; Gremel et al., 2016). Technically, SIP is classified as an “adjunctive” behavior along with other behaviors reliably produced by schedules (for reviews see Flory, 1971; Roper, 1978; Singer et al., 1982). Adjunctive or displacement behaviors are considered a separate behavioral class outside of those produced by classical operant or Pavlovian learning paradigms, and are incentive in nature (Breland and Breland, 1961). Attempts to re-classify SIP as partially goal-directed in nature highlights the elements of the behavior that overlap with operant mechanisms (Killeen and Pellón, 2013). SIP expression is contingent on multiple pairings of salient stimuli in a defined context (Falk, 1966, 1967; Flory, 1971; Lashley and Rosellini, 1980) suggesting acquisition and expression of excessive drinking engages classical learning processes (Hawken and Beninger, 2014). Incidentally, initial SIP expression is likely the result of normal goal-directed learning that over time, *can* precede an over reliance on habitual responding observed in compulsivity (Hawken and Beninger, 2014) (for review see Gillan and Robbins, 2014).

The most commonly reported brain abnormality in OCD and OCSD is dysregulation of the neural feedback circuit that involves both goal-directed and habit circuits, including frontal, limbic, and striatal structures (Saxena et al., 1998; Graybiel and Rauch, 2000; Kopell and Greenberg, 2008). As in humans, compulsive-like animals may have an inherent predisposition to form habits and compulsive behavior due to imbalanced striatal circuits (Graybiel and Rauch, 2000; Gillan et al., 2011). In SIP, we demonstrated that animals who use predominately striatal-learning strategies (those that rely on the integrity of the striatum – specifically the DLS) (Packard and McGaugh, 1996) to learn the location of a food pellet in a Y-maze drink significantly more water when subsequently exposed to the SIP paradigm (Gregory et al., 2015). In addition, spine density in DLS neurons was found to increase following SIP demonstrating that plasticity in brain regions central to habit formation may contribute to SIP (Íbias et al., 2015). Furthermore, early in SIP training we found significant increases in neuronal activation in the mPFC and OFC regions. The increase in immediate early gene (IEG) FosB/ΔFosB was most pronounced in animals that demonstrated both SIP and striatal-learning tendencies. Repeated activation of OFC-ventral striatal pathway in mice produced grooming sensitization over days further supporting cortical-striatal involvement in compulsive behaving (Ahmari et al., 2013). Additionally, SIP-prone rats exhibited more c-Fos activity in the OFC and basolateral amygdala compared to non-compulsive drinking animals (Merchán et al., 2018). This is in line with human studies that show increased metabolic activity of the striatum and OFC in OCD patients and during symptom provocation (Breiter and Rauch, 1996; Saxena et al., 1999). However, the identity of the discrete mechanism(s) in the OFC/subcortical regions responsible for selecting appropriate behavior via goal-oriented or habit circuit recruitment in humans is not fully known.

## The Bed Nucleus of the Stria Terminalis (BNST) in Homeostatic Emotion and Compulsive Behaving

Current effective treatments for OCDS may provide further insight into putative neurophysiology. Deep brain stimulation of the BNST has yielded sustained symptom relief in obsessions and compulsions in otherwise refractory OCD (Nuttin et al., 1999, 2013; Raymaekers et al., 2017; Winter et al., 2018). A collection of several sexually dimorphic interconnected nuclei, the BNST is part of the extended amygdala with extensive bi-directional connectivity with the CLSTC-loop and beyond (Prewitt and Herman, 1998; McDonald et al., 1999; Dong et al., 2001; Hasue and Shammah-Lagnado, 2002; Dong and Swanson, 2003, 2004b, 2006a,b,c; Rodaros et al., 2007; Li and Kirouac, 2008; Avery et al., 2014; Gregory et al., 2019). Functionally, the BNST filters and/or integrates multiple ascending modalities, mapping with adequate resolution, interoceptive information onto motivational systems for adaptive physiological and behavioral outcomes (Jennings et al., 2013; Kim et al., 2013; Lebow and Chen, 2016). Preclinical work compliments studies in humans: we have demonstrated a role for the oval subregion of the BNST (ovBNST) in compulsive behaviors including addiction, compulsive sucrose-seeking, and finally, in SIP (Krawczyk et al., 2013; Gardner Gregory, 2018; Maracle et al., 2019). Accordingly, electrical stimulation of the BNST is reported to suppress SIP in rodents (van Kuyck et al., 2008). Together, these findings highlight an emergent player, the BNST, in compulsive neurophysiology.

The BNST is fast becoming a relevant region of interest in stress-related psychiatric illness because of its role in emotion processing (Avery et al., 2016; Lebow and Chen, 2016). OCD is “stress responsive,” with stressful events precipitating OCD onset and symptoms (Toro et al., 1992). Nuclei within the BNST are bi-directionally connected to several nuclei in both the hypothalamus and the amygdala (Dong et al., 2001; Dong and Swanson, 2004a,b; Jennings et al., 2013), two structures implicated in stress and anxiety, respectively. A role for the cortico-amygdalar circuitry in OCD is emerging (Simon et al., 2014), however, how the hypothalamic-pituitary-adrenal (HPA)-axis, a key region involved in stress-reactivity, and stress itself impacts OCD pathophysiology is understudied. Along the HPA-axis in rodents, adrenalectomy hastens the emergence of SIP and exogenous corticosterone reverses the effect (Table 2; Devenport, 1978). In intact rats, corticosterone administration also inhibits SIP (Tang et al., 1984; Mittleman et al., 1988b). Furthermore, anxiogenic stimuli (e.g., foot shock) that increase corticosterone also suppress SIP acquisition (Brett et al., 1982). However, foot shock effects on SIP are dose-dependent, where low shock intensity augments SIP and high voltage prevents SIP (Galantowicz and King, 1975). Together, these findings indicate stress reactivity may modulate SIP and/or compulsivity. Given its role in processing classic emotional stimuli and events (e.g., stress and anxiety), we further postulate that the BNST encodes homeostatic emotions, specifically hunger and satiety, to guide appropriate adaptive responding.

**FIGURE 2:**
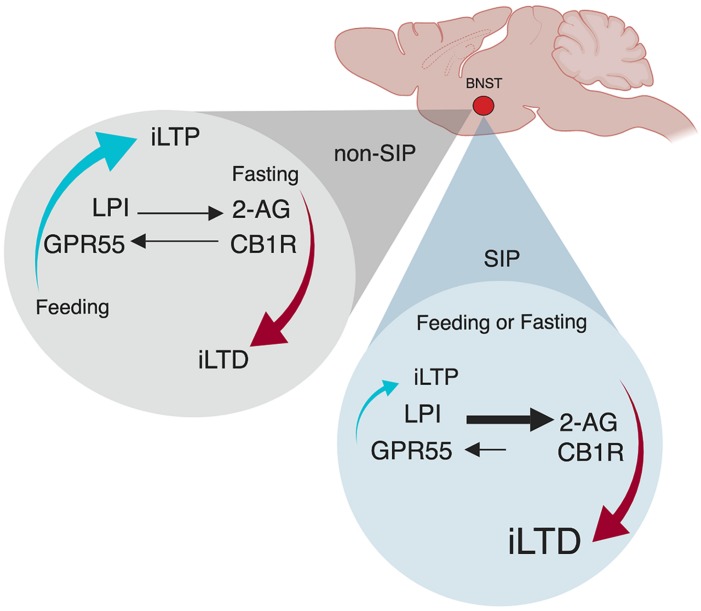
Oval bed nucleus of the stria terminalis (ovBNST) inhibitory plasticity is satiety-state independent in SIP. Adapted with permission from Hawken et al. (2019) and Gardner Gregory (2018). Plasticity at ovBNST GABAergic synapses is caloric-state dependent exhibiting bi-directional plasticity. In a sated state, synapses are biased toward iLTP but when food deprived, they can also express iLTD. When refed, synaptic plasticity quickly returns to iLTP. However, in SIP animals, refeeding does not reinstate iLTP, the mechanism becomes stuck as bidirectional plasticity is lost in animals that show compulsive behaving. iLTP, inhibitory long-term potentiation; iLTD, inhibitory long-term depression; LPI, L-α-lysophosphatidylinositol GPR55 ligand; GPR55, putative cannabinoid receptor; 2-AG, 2-arachidonoylglycerol CB1R ligand; CB1R, cannabinoid receptor.

Indeed, we recently identified a neural mechanism within the ovBNST that may represent the homeostatic emotion of hunger-satiety (Figure 2; Hawken et al., 2019). In order to establish compulsive drinking through the SIP protocol, animals must first be hungry, a drive-state typically induced through food restriction (Figure 1). In sated (fed *ad libitum*) male rats, low-frequency stimulation of ovBNST GABAergic synapses produces increases in inhibitory postsynaptic currents and promotes long-term potentiation (iLTP) in the majority of neurons recorded. Following acute food-restriction (24 h), however, GABAergic plasticity toggles to long-term depression (iLTD) via endocannabinoid dependent mechanisms (Figure 2; Hawken et al., 2019). In this framework, the novel endocannabinoid receptor GPR55 and its ligand, LPI, mediate a hunger-satiety signal (iLTP) while the classic endocannabinoid CB1 receptors and their ligand, 2-AG, promote a hunger-state (iLTD). This effect is plastic as after a brief refeeding window, iLTP is reinstated. In SIP-prone rats, bi-directional inhibitory plasticity in ovBNST neurons is lost, unable to toggle between iLTP and iLTD, as synapses become stuck transmitting a “hunger” signal well after being refed (Figure 2; Gardner Gregory, 2018). In this case, CB1 receptors and 2-AG drive reduced GABA transmission in the synapse and iLTD predominates. A loss of synaptic plasticity may be reflected in SIP studies that confirm SIP-prone rats continue to excessively drink despite correcting for their caloric deficit (Falk, 1971). Thus, synapses within the ovBNST driven by hunger and satiety cues lose the ability to correctly encode representative homeostatic emotion. Future research is needed to explore the consequences of inflexible GABAergic synapses in the ovBNST in the bottom-up development of complex behaviors like compulsivity.

A role for the BNST in compulsivity, however, is gleaned by the structural and functional connectivity of its nuclei within the CLSTC-loops implicated in compulsivity. Locally, the ovBNST subregion sends projections to the lateral hypothalamus with efferents and afferents to regions of the amygdala (Dong et al., 2001). The ovBNST also is bi-directionally connected to the fusiform (Dong et al., 2001), another nucleus within the BNST complex thought to receive projections from the frontal cortex (Lebow and Chen, 2016). Top-down, structural and functional projections from the frontal cortex (i.e., OFC/PFC/insula) to the BNST have been identified in humans, non-human primates, and rodents (Reynolds and Zahm, 2005; Fox et al., 2010; Motzkin et al., 2015). Bottom-up, pathways from the BNST to the frontal cortex are likely multi-synaptic and indirect via the amygdala, striatum, or other areas. For instance, the BNST in humans and rodents is both structurally and functionally connected to the NAc (Dong and Swanson, 2004b; Wood and Swann, 2005; Avery et al., 2014). Both the BNST and the NAc have prominent roles in compulsive drug use (Koob and Volkow, 2010, 2016; Krawczyk et al., 2013). Consistent with a distinct role in “valence surveillance” (Lebow and Chen, 2016), we postulate that the BNST, in part, contributes bottom-up afferent interoceptive information regarding homeostatic satiety via direct and indirect connections with the cortex and goal-directed and habit circuits to represent and convey homeostatic emotions of hunger and hunger-satiety.

As a candidate for encoding homeostatic emotions, the extended amygdala (i.e., the BNST) acts as an integrative hub to detect and signal exteroceptive and interoceptive shifts in the body. Subsequently, the BNST transduces homeostatic information onto circuits that assess contexts and invigorate behaviors to correct any physiological imbalance (eating when hungry, drinking when thirsty or escape/avoidance of threat stimulus). Thus, inaccurate neural representation of interoceptive information within the BNST could have drastic behavioral impact. For instance, the reinstatement of bi-directional GABA plasticity (i.e., iLTP) in the ovBNST following hunger-satiety could signal to the cortical structures a need to shift responding strategies to stop food-seeking behaviors. When iLTD persists despite homeostatic correction (feeding), cortical structures receive a “hunger” signal and continue to engage circuits that promote preparatory and consummatory behaviors, including locomotion and drinking. Convergent findings from animal and human research postulate that together with the mPFC (Killcross and Coutureau, 2003), activity of the OFC manages the activity of subcortical pathways including the striatum (Gremel and Costa, 2013; Pauls et al., 2014; Gremel et al., 2016). Perhaps based on the firing frequency of glutamatergic inputs to the OFC, the OFC selects the appropriate circuit to activate (either goal-oriented or habit) downstream from the cortex (Gremel et al., 2016). In the presence of increased striatal DA reactivity, circuits may be weighted for preferential activation by cortical structures, resulting in a loss of striatal circuit plasticity. In this way, inadequate resolution of homeostatic emotion by subcortical structures through aberrant neural plasticity may promote maladaptive behavioral responding ultimately dictated by the OFC (Hardung et al., 2017).

## Conclusion

Using compulsive drinking in humans (primary polydipsia) and rodents (schedule-induced polydipsia) we suggest a putative neurobiological framework for the etiology of compulsive behaviors. Thirst, hunger, and satiety constitute essential homeostatic emotions, or subjective feelings that drive motivated, goal-directed behavioral selection needed for survival. In polydipsia, homeostatic emotions fail to illicit appropriate adaptive behaviors. How the brain might switch from a state responsive to shifts in homeostasis to one that is unresponsive was surmised by identifying key over-lapping brain changes between polydipsia associated with schizophrenia, schizophrenia without polydipia, and obsessive compulsive disorder. Composite data suggest that DA-regulated neural plasticity within the striatum and regions of the cortex underlie compulsive phenotypes. Ecological and validated animal models of compulsive drinking, or SIP, confirm a role for increased striatal DA activity in compulsive behaving. Additionally, literature points toward aberrant (non-exclusive) monoamine (e.g., DA) modulation of goal-directed and habitual behavior within cortico-limbic-striato-thalamic-cortical loops. Exciting new evidence suggests that non-representative encoding of homeostatic emotions (hunger/satiety) by a cluster of nuclei in the extended amygdala, promote the development of compulsions observed in SIP. The BNST’s role in valence attribution or “valence surveillance” and its extensive web of connectivity within the cognitive, affective, and reward-related circuits highlights the importance of accurate encoding and bottom-up (to the cortex) transduction of homeostatic emotions. Aberrant neuroplasticity within bottom-up and top-down circuits that support homeostatic emotion signaling likely contribute to maladaptive behavioral selection and inappropriate responding. Future research can explore our postulated mechanism of compulsive behaving.

## Author Contributions

TB and EH equally contributed to the design, writing, and editing of the manuscript.

## Conflict of Interest Statement

The authors declare that the research was conducted in the absence of any commercial or financial relationships that could be construed as a potential conflict of interest.
